# Economic Conditions Predict Prevalence of West Nile Virus

**DOI:** 10.1371/journal.pone.0015437

**Published:** 2010-11-12

**Authors:** Ryan J. Harrigan, Henri A. Thomassen, Wolfgang Buermann, Robert F. Cummings, Matthew E. Kahn, Thomas B. Smith

**Affiliations:** 1 Center for Tropical Research, Institute of the Environment, University of California Los Angeles, Los Angeles, California, United States of America; 2 Institute of the Environment, University of California Los Angeles, Los Angeles, California, United States of America; 3 Orange County Vector Control District, Garden Grove, California, United States of America; 4 Department of Ecology and Evolutionary Biology, University of California Los Angeles, Los Angeles, California, United States of America; University of California, Berkeley, United States of America

## Abstract

Understanding the conditions underlying the proliferation of infectious diseases is crucial for mitigating future outbreaks. Since its arrival in North America in 1999, West Nile virus (WNV) has led to population-wide declines of bird species, morbidity and mortality of humans, and expenditures of millions of dollars on treatment and control. To understand the environmental conditions that best explain and predict WNV prevalence, we employed recently developed spatial modeling techniques in a recognized WNV hotspot, Orange County, California. Our models explained 85–95% of the variation of WNV prevalence in mosquito vectors, and WNV presence in secondary human hosts. Prevalence in both vectors and humans was best explained by economic variables, specifically per capita income, and by anthropogenic characteristics of the environment, particularly human population and neglected swimming pool density. While previous studies have shown associations between anthropogenic change and pathogen presence, results show that poorer economic conditions may act as a direct surrogate for environmental characteristics related to WNV prevalence. Low-income areas may be associated with higher prevalence for a number of reasons, including variations in property upkeep, microhabitat conditions conducive to viral amplification in both vectors and hosts, host community composition, and human behavioral responses related to differences in education or political participation. Results emphasize the importance and utility of including economic variables in mapping spatial risk assessments of disease.

## Introduction

Understanding the environmental conditions that lead to infectious disease outbreaks, though challenging, is often crucial for management and control [1]. The recent introduction of West Nile virus (WNV) to North America serves as a poignant reminder of the impact that a novel infectious disease may have on a naïve biological community. In the last decade, WNV has infected numerous vector and host species [2]–[4], leading to continental-wide declines in bird populations [5], [6], an estimated 3 million infections [7] and thousands of deaths in human hosts, and the expenditure of millions of dollars on control and vaccination efforts [8]. These efforts have focused on disease “hotspots”, where WNV-infected vectors (mosquitoes), primary hosts (birds), and secondary mammalian hosts (including humans) co-occur in close proximity. Predictors such as natural ecological conditions [9], [10], urbanization or other anthropogenic factors [10], [11], and land cover characteristics [9], [12], have all been associated with human WNV hotspots. A number of mechanisms can contribute to disease outbreaks, including higher absolute pathogen prevalence, increased density of vectors and hosts, or increased rates of transmission due to variation in host behavior. The seemingly unpredictable nature of localized outbreaks and the fact that high WNV incidence has been reported from across a broad range of ecological conditions [12]–[15] has made forecasting WNV hotspots virtually impossible [16], [17].

Orange County, California, is a recognized hotspot of WNV, having recorded positive mosquito and human cases every year since 2004 and the third highest number of reported human cases per county in the United States in 2008 (the Southern California area reported 4 out of the 5 highest number of WNV human cases per county this same year, USGS, http://diseasemaps.usgs.gov). The region comprises an area of approximately 40×60 km and is characterized by spatially variable ecological conditions, including shallow valleys that have been dramatically altered by urbanization. The result is a heterogeneous topographic and economic landscape comprised of high-income communities in valleys and coasts, and lower-income communities within major urban centers. Recent financial downturn has led to a rise in both the number of home foreclosures and neglected (unchlorinated) swimming pools [18]. During the primary months of WNV activity (May-October), the entire study region experiences high average daily temperatures and little to no rain. As a result, vectors, and the hosts on which they feed, must rely on natural and artificial standing water sources. While several mosquito species have been identified as WNV vectors in Southern California, the dominant WNV vector in Orange County is the Southern House mosquito, *Culex quinquefasciatus* Say, which is an important vector of WNV [19] and other mosquito-borne pathogens across much of the southern United States [20], Hawaii [21] and Central and South America [22].

Here we investigate WNV prevalence in vector populations and secondary human hosts in Orange County, California, between 2004–2008, using a combination of machine learning algorithms and spatially explicit ecological modeling. To best capture conditions that might be associated with WNV incidence, we used a diversity of predictors, including both ecological and economic variables. We attempt to accurately predict future prevalence hotspots in both vectors and hosts using observed prevalence levels and their relationship to the current environmental and economic landscape.

## Results

Using a combination of economic and environmental variables and various machine learning algorithms, we were able to explain a significant proportion of variation in WNV prevalence in vectors, and accurately predict both prevalence in vectors, and disease incidence in human hosts. The explained variation in WNV prevalence in vectors was highest in 2008, with a maximum of 95% of the variation explained and a root mean square error (RMSE) of 9.6% of the total variation (Table 1). Models for years 2004 and 2005 also explained much of the observed variation (2004: 85%, RMSE of 14%, 2005: 92%, RMSE of 11.2%), while prevalence levels for 2006 and 2007 were too low for statistical analysis. In attempting to predict test data not used in model construction, we were able to explain a maximum of 56% of the variation in WNV prevalence in 2008, 6% in 2004, and 34% in 2005.

**Table 1:** Relationship between observed versus predicted values of WNV vector prevalence under random forest models using the same data set for testing and training, and under models where ∼36% of the data was separated from training and used as test data (36).

	2004	2005	2008
Single Model	**88%** (14%)	**92%** (11.2%)	**95%** (9.6%)
Separate Testing and Training Models	**6%**	**34%**	**54%**

Root mean square error (RMSE) percentage of observed prevalence range are reported for single models (parentheses), whereas out-of-bag (OOB) error rates were used in calculations of percent variation explained in the separate random forest models.

**Figure 1:**
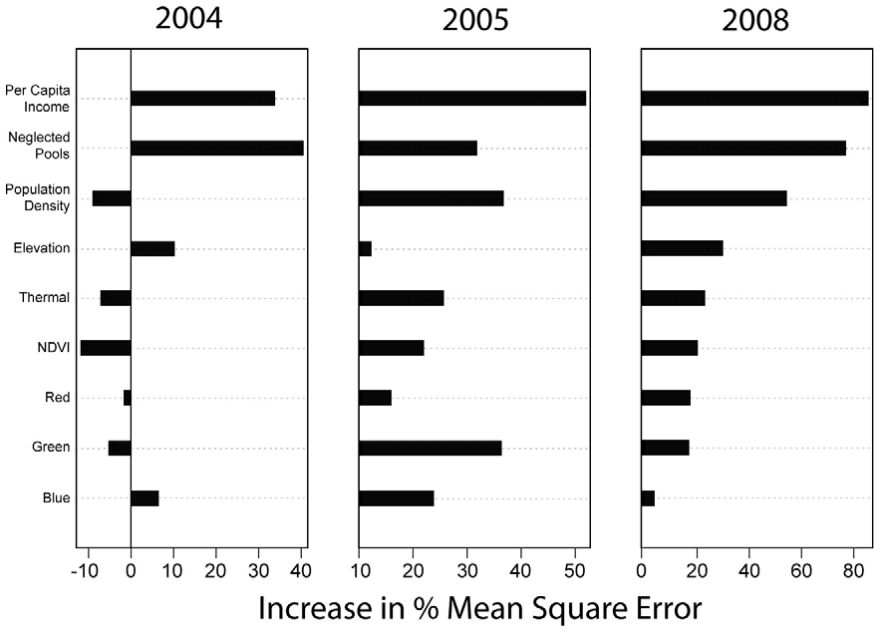
Variable importance scores under random forest models for both ecological and economic predictors of West Nile virus prevalence in a West Nile virus hotspot. Percent mean square error indicates the increase in error in out-of-bag samples when that variable is permuted, with higher increases indicative of more important variables. Negative changes in mean square error percentage (2004) suggest that random permutations of a variable perform better under random forest than actual values, indicating a poor predictor. There were not enough West Nile virus positives in vectors for years 2006 and 2007; thus, these years were excluded from analyses.

**Figure 2:**
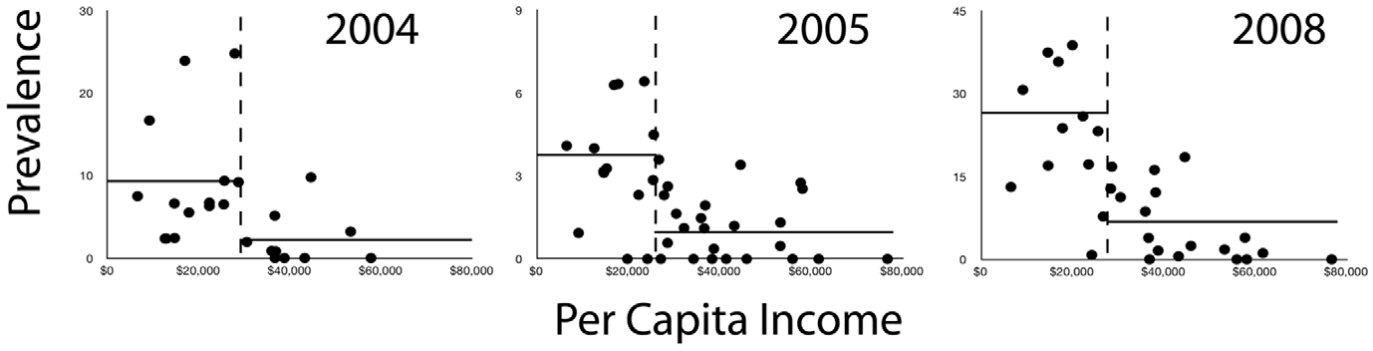
Relationship between average per capita income and West Nile virus prevalence. Results are shown for vectors in Orange County, California, for 2004, 2005, and 2008. Prevalence is measured as MLE. Dashed lines indicate the bifurcation between high and low prevalence values as determined by tree regressions. Horizontal lines indicate mean values of prevalence for points above and below this bifurcation (Wilcoxon rank-sum tests for these means were significant for each year, p<0.001). Although absolute measures of WNV prevalence varied between years, relationships between predictors (per capita income in this case) and WNV prevalence were stable throughout the study period.

Across all years, economic variables explained the largest amount of variation in WNV prevalence in vector populations (Figure 1). Per capita income was the most important predictor variable, and was a significant contributor to all random forests explaining the spatial distribution of prevalence of WNV in vectors (Figures 1, S4). Inspection of the relationship between per capita income and WNV prevalence also revealed that higher prevalence levels in vectors were consistently associated with lower-income areas (Figure 2).

Previous research indicates that both natural and artificial water sources may play a role in determining West Nile virus incidence levels [23]–[25]. We found that, in addition to per capita income, the density of neglected swimming pools within 1 km of observed prevalence was a particularly important variable in years with high WNV prevalence. The region receives little rainfall during the months of WNV activity (Figure S2), underscoring the likely role of artificial neglected pools acting as potential vector breeding locations. During the study period, the county experienced a rise in the number of foreclosed homes and neglected pools [18]. This suggests that neglected swimming pools may promote WNV amplification, and may represent a direct link between declining economic conditions and a favorable environment for WNV propagation.

**Figure 3:**
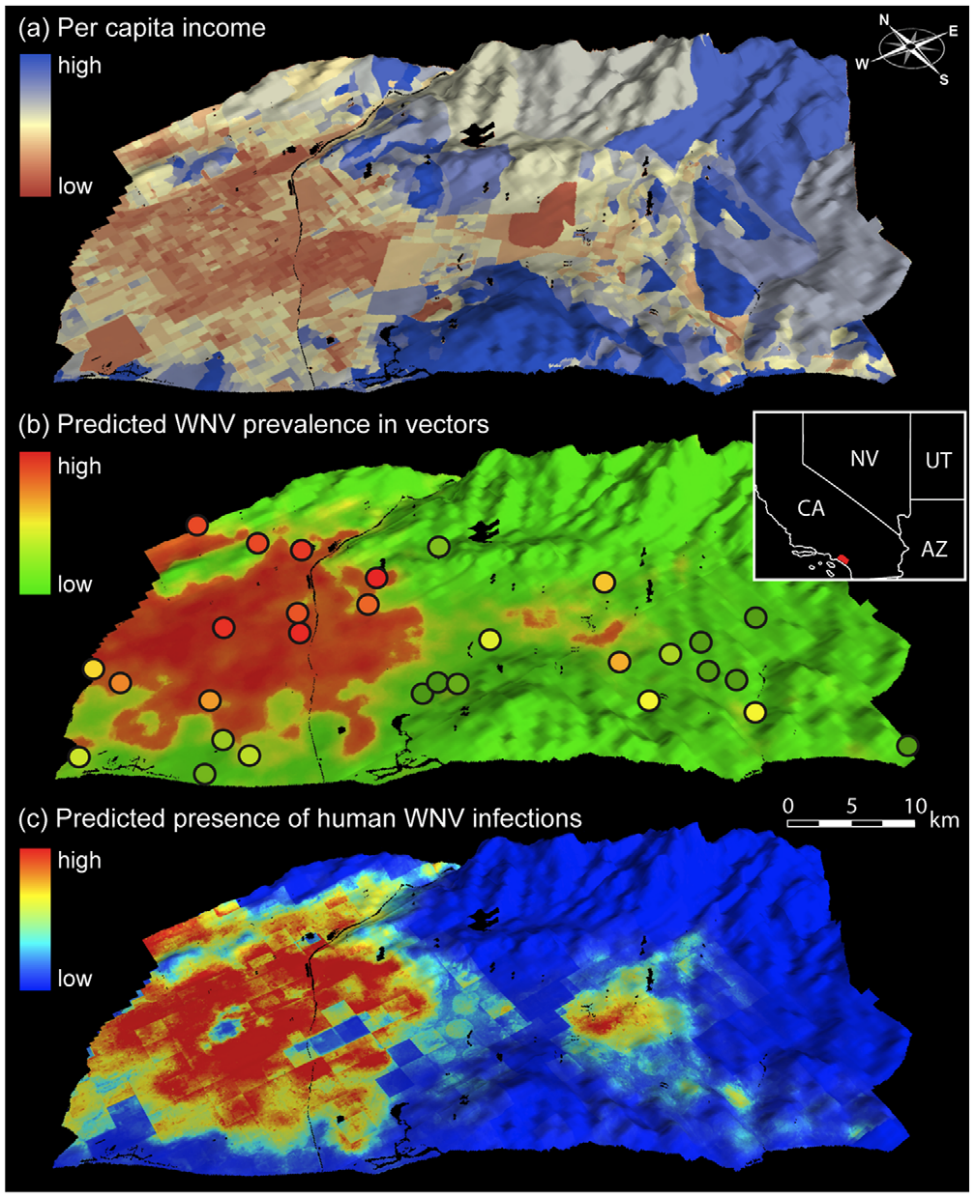
Spatial predictions of West Nile virus in vectors and human populations. (A) Data layer representing per capita income across the county, as collected as part of the 2000 U.S. National Census. (B) Predictions of WNV prevalence in vectors across the study area for 2008 based on the 2005 WNV prevalence model. Circles indicate observed WNV prevalence levels in 2008 using the same color codes. (C) Predictions of WNV presence in human hosts in 2008 across the study area, determined using niche modeling (Maxent; *25*). Scale bar is an approximation, as scale varies according to perspective.

Spatially continuous interpolation of WNV prevalence across the study area predicted strong heterogeneity in WNV prevalence, with low-income, densely populated areas showing higher prevalence of WNV in vector populations. Although the highest observed values of WNV prevalence varied between years (Figure 2), the relationship between lower per capita income and higher prevalence of WNV in vectors remained constant across years (Figure S5). This permitted predictions of future WNV prevalence using only data from previous seasons. For example, models constructed using 2005 prevalence data were able to explain 52% of the observed prevalence variation in 2008, with consistent WNV hotspots across years (Figure 3).

Using niche modeling to capture presence of WNV in human hosts, predictive models performed well (AUC_2004_  = 0.951; AUC_2005_  = 0.933; AUC_2008_  = 0.940), detecting a significant relationship between human WNV infections and environmental and economic variables. Jackknifing of predictor variables showed that for 2004, per capita income was the single best variable for model performance, and resulted in the largest decrease in model performance when omitted (Figure S6). Under the same criteria, density of neglected swimming pools and per capita income were the most important variables in explaining variation in 2005 and 2008. Spatial predictions identified areas of high incidence of WNV in secondary human hosts, which closely corresponded to those representing high prevalence in WNV vectors (Figures 3, S6). In sum, WNV presence in both vector and secondary human host populations can best be explained using the same predictor criteria, suggesting a common ecological mechanism driving viral occurrence.

## Discussion

We found that economic conditions best explain WNV hotspots. By documenting the occurrence of this widespread, recently introduced virus in vector and host populations, we identified the correlates of disease occurrence and how these can be used in forecasting future outbreaks. While predictors of West Nile virus are likely to vary with local conditions [26], [27], our results consistently show that lower income areas represent habitats conducive to West Nile virus amplification in vectors, leading to higher occurrence of the disease in secondary human hosts.

West Nile virus prevalence in vectors may be higher in lower-income communities for at least three reasons. First, densely populated areas generally occur on flatlands at lower elevations, characterized by older infrastructure with antiquated water runoff systems. These factors could contribute to poor drainage and favorable mosquito breeding habitats [28]. However, elevation was an important variable in predicting WNV prevalence in only one year of the study, and never contributed more to explaining prevalence than did economic variables. Second, lower-income communities are likely to invest less in private property upkeep. This is supported by our data showing an increase in prevalence with higher densities of neglected pools. Neglected swimming pools were a significant predictor across years, and provide evidence that untreated artificial water sources associated with a rise in home foreclosures [18] may promote an increase in outbreaks of West Nile. Finally, income is positively correlated with education, and better-educated people are more likely to be politically engaged in demanding pest control services, such as mosquito management [29].

Each of the possible factors above imply that higher prevalence of West Nile virus in lower-income communities can simply be attributed to more vector breeding sites or higher abundances of vectors in these areas. However, there were no significant associations between vector abundance and economic conditions in our study (See Methods). This suggests that lower-income communities represent complex ecological microhabitats conducive to viral amplification in vectors and hosts, rather than just locations conducive to increased vector breeding. Enzootic cycles of West Nile virus can be affected by numerous factors, including temperature [30], precipitation [24], and vector and host heterogeneity [27], [31]. Higher avian diversity has been associated with lower occurrence of WNV in a variety of habitats [27], [32]. As a result, a loss of primary host diversity may lead to increased prevalence of West Nile virus, particularly when remaining species are effective reservoir hosts. In Orange County, mosquito blood-meal analysis [33] suggests that House Finches and House Sparrows are the most frequently-fed upon, competent hosts of West Nile virus, both of which are highly abundant in urban habitats and have previously been implicated in playing important roles in WNV amplification and transmission [34]–[36] in California. Further, open water sources, such as neglected swimming pools, may bring WNV vectors and hosts in close proximity, aiding in disease transmission [24]. Southern California, in particular Orange County, is characterized by urban development that includes extensive housing tracts of small single-family homes in lower-income areas, many of which have swimming pools. Although most homeowners are above the poverty level, amenities such as swimming pools may not receive regular upkeep as economic conditions worsen. Neglected pools have the potential to become eutrophic water bodies capable of supporting high densities of immature mosquitoes. These factors, combined with microhabitat ecological conditions particularly suitable for the primary vector, *C. quinquefasciatus*, may be responsible for the elevated infections of West Nile virus observed in vectors and human hosts in lower-income areas.

While the conditions leading to West Nile outbreaks have proven difficult to determine, the observed power of economic variables in predicting the impacts was remarkable. In this regard, it is unlikely that Orange County is unique; in fact, high incidences of West Nile virus have recently been attributed to both urbanization [10] and the homogenization of landscapes and avian communities [32], [37], [38] across diverse environments. Considering these trends, WNV is likely to continue to pose public health risks in urban areas. Our findings demonstrate the importance of including economic factors in predicting future outbreaks and emphasizes the need for additional research into the specific ecological variables that may be driving these patterns.

## Materials and Methods

For vector data collection, batches of mosquitoes (hereafter referred to as mosquito pools) were sampled from 2004–2008 throughout Orange County by the Orange County Vector Control District (OCVCD). In each year, sampling sites were distributed across Orange County, with a core of ∼20 sites where data were collected yearly, and an additional 5–10 satellite sites that were sampled during a single year. Mosquitoes were collected from May-October using either CDC-style light traps [39] intended to capture a wide variety of potential WNV vectors (primarily mosquitoes in the *Culex* genus) or gravid traps [40] that were specifically baited to capture the most prominent WNV vector in the area (*C. quinquefasciatus*). For each mosquito pool we calculated WNV prevalence as the Maximum Likelihood Estimate (MLE) [41] of mosquitoes positive for WNV. MLE is often regarded as a more accurate measure of prevalence than the percentage of infected mosquito pools, because it accounts for the possibility that more than a single mosquito is infected per tested mosquito pool. While more recent vector-based surveillance measures have been implemented in an attempt to dissect the contribution of individual vectors to WNV spread [26], [42], we used only a measure of infection rate (as measured by MLE) as a representative of WNV prevalence levels, for the following reasons: 1) comprehensive measures of vector abundance (a requirement for determining relative importance of each WNV-positive vector) were not recorded for years 2004 and 2005, 2) for years in which these abundance measures were taken (2006–2008), spatial heterogeneity of vector abundance did not vary dramatically across our study period (no more than an order of magnitude, in comparison to several orders of magnitude in previously studied systems), 3) WNV transmission is likely driven by a single dominant vector, *C. quinquefasciatus*, as evidenced by a relatively homogenous vector community composition. During the year (2008) in which abundance data was methodically collected and WNV prevalence was high, measures of Vector Index and MLE values were highly correlated (Adj. R^2^ = 0.69, *p* = 1.3×10^−8^). In addition, the overlap in prediction areas based on our MLE estimates and the human cases reported in our study area (see Results, Figures 2, S5) suggests that our measures of vector prevalence are successfully capturing the risk of WNV infections to secondary hosts. Only sites that were sampled ≥15 times during the WNV season (defined as weeks 18 through 43 of each year) were used for analyses. In addition, to ensure that our sampling effort was spatially and temporally unbiased, we checked for both spatial autocorrelation (see supporting information Methods Text S1, Table S1, Figure S3), and continuous distribution of sampling throughout the season, with no obvious clusters of sampling dates, or gaps in sampling effort. West Nile virus prevalence levels were too low in years 2006 and 2007 to establish statistically meaningful relationships; for this reason we restricted our analyses to years 2004, 2005, and 2008.

Locations of confirmed infections of WNV infections in humans in 2004 (n = 61), 2005 (n = 17), and 2008 (n = 75) were compiled and provided by the Orange County Health Care Agency (OCHCA). These confirmed infections included WN neuroinvasive disease, WN fever, and positive blood donors. OCHCA only included cases for which infections most likely occurred in Orange County, and omitted those that may have occurred outside the county. For privacy considerations, random spatial error was introduced (less than 1 km) to the locations by OCHCA. Because the introduced error was random and within the nightly ranges of primary WNV vectors, it is unlikely that this procedure affected any analyses, especially in any particular direction. We are therefore confident that the associations presented here are not the result of data treatment.

To assess the influence of environmental heterogeneity on WNV prevalence in a GIS-framework, we used a set of environmental variables comprising raw measurements and derived products from the Advanced Spaceborne Thermal Emission and Reflection Radiometer (ASTER) instrument. From ASTER images taken in May 2005, we used the red, green, and blue visible bands as individual variables, as well as the thermal band, which measures surface kinetic temperature. We used images from May, as this is the first month for which positive West Nile virus samples were used for analyses. Analysis of spatial heterogeneity in temperatures for three years in Orange County, California, at nine ground stations (provided by the Ames Research Center and collected through the National Climatic Data Center, NCDC, www.ncdc.noaa.gov) suggested that temperature was homogenic across the study area, and more importantly that patterns of heterogeneity did not change over the course of summer months (Figure S1). Measures of precipitation taken from these nine ground stations from May-October (the study period for each year) were minimal (Figure S2), suggesting that natural precipitation levels or spatial heterogeneity in precipitation does not contribute appreciably to explaining variation in WNV prevalence during these months. Precipitation measurements as summarized in layers made available by the WorldClim group [43] corroborated the finding that there is little spatial heterogeneity in our study area.

Visible ASTER bands were used at the native resolution of 15 m, and the thermal band was reaggregated to the same grid cell size, while retaining its native 90 m resolution. In addition, Normalized Difference Vegetation Index (NDVI), a vegetation index that correlates well with plant leaf density in most environments, was computed through the normalized difference in surface reflectances at near-infrared (NIR) and red wavelengths. This was derived by first atmospherically correcting ASTER bands 2 (red) and 3n (IR) using the software ATCOR 2 (Atmospheric Correction for Flat Terrain, ReSe Applications Schläpfer) with the default coefficients, in order to obtain accurate surface reflectance values, and then applying the equation: 
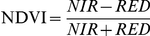



Previous studies suggested a relation between WNV prevalence and the presence of standing natural and artificial water sources [11], [23]–[25], and because precipitation was minimal during study period months, we also included a layer capturing the density of neglected swimming pools in our analyses. Neglected pools were defined as untreated (un-chlorinated) pools that could be potential breeding sites for mosquitoes, and were identified by OCVCD through a combination of aerial surveys and ground-truthing. The total number of neglected pools for 2004, 2005, and 2008 was 417, 448, and 1,428 respectively. For each 15×15 m gridcell, we calculated the density of neglected pools within a 1 km radius, an estimate within the nightly range of relevant mosquito species [44].

As natural standing water bodies may also act as potential breeding sites for mosquitoes, and because we expected elevation to affect the amount of standing water available to vectors and hosts (lower valley areas are hypothesized to have more runoff and standing water pools than higher elevation sites), we included a 25 m resolution elevation value for all sites surveyed in analyses. We obtained this layer from the ASTER Global Digital Elevation Model data set made available free-of-charge from a joint venture between the Ministry of Economy, Trade, and Industry (METI) of Japan and the United States National Aeronautics and Space Administration (NASA) (http://www.ersdac.or.jp/GDEM/E/).

To assess the role of heterogeneity in economic conditions on WNV prevalence, we extracted ‘mean population density’ and ‘per capita household income’ from Census 2000 data available at the California Spatial Information Library (http://casil.ucdavis.edu/casil/society/) and US Census Bureau (http://www.census.gov). ‘Per capita household income’ is generally considered to be a good descriptor of the economic situation in residential areas. Although more recent socioeconomic data are available, we used the data from 2000, because these data are at the highest resolution currently available (block group level, containing 600–3000 people, with an optimum of 1500). Potential caveats of using data summarized at the census block level are that the sizes of block groups vary, and that potential sharp transitions of socioeconomic measures among block groups may not reflect the true socioeconomic and related environmental conditions at block group boundaries. In addition, even when the recorded sharp transitions among block groups are accurate, prevalence levels of WNV in these boundary areas may be influenced by proximity to different socioeconomic or environmental conditions. To reduce the potential for drawing incorrect conclusions due to these limitations and boundary effects, we computed the average ‘mean population density’ and ‘per capita household income’ within a radius of 1 km, effectively resulting in layers with smoothed transitions among block groups.

Processing of spatially explicit data was carried out in ArcMap 9.2 (ESRI, Redlands, CA). Economic GIS data layers were converted from feature data to raster grids. Because the modeling software packages require that input data layers are in the same format, all GIS data layers were reaggregated to 15 m grid cell resolution, corresponding to that of the visible band layers derived from the ASTER instrument.

To model prevalence levels of WNV in vector pools across the study area, we used a suite of economic (per capita income, human population density, and density of neglected swimming pools) and ecological (vegetation, temperature, and topography) variables in tree regression [45] and random forest models [46] in the R statistical framework [47] to assess the relative importance of each variable in predicting WNV prevalence in vectors. Regression tree models implement binary recursive partitioning procedures to measure the amount of variation in a response explained by each predictor used in the model. No *a priori* assumptions are made about the relationship between predictor and response variables, allowing for the possibility of non-linear relationships with complex interactions. The resulting bifurcation is presented as a tree in which the nodes represent the predictor variables that split the response variable data set into two partitions, such that the homogeneity within each partition is maximized (see supporting information Methods Text S1, Figure S4). Homogeneity is measured by the Gini index [46], and splitting continues until further partitioning does not reduce the Gini index. The length of the branches following each partition indicates the relative importance of the partitioning predictor variable. Unlike typical regressions, these non-linear, non-parametric functions can indicate the comparative amount of variation explained by each variable [48], [49]. Random forests represent iterations of regression trees, where both records and predictor variables are randomly permuted to assess the robustness of classifications found. These permutations include bagging procedures [50], [51] where a random subsample of the original dataset is taken to construct regression trees. The samples that are not included in the random subsample - the out-of-bag samples - are subsequently used to test the model predictions from the bagged samples. These methods also incorporate a randomization of predictor variables used to construct each of the numerous regression trees [51]. The iterative nature of these models provides statistically rigorous statements about the relationships between predictor and response variables, as measured by the percent of variation explained by the full forest, and by measures of individual variable importance [48], and results have been shown to outperform traditional regression techniques [49], [52]. In our analyses, environmental and economic data values corresponding to the grid cell at the sampling locations were extracted, and 2000 iterations were run with ∼36% of the samples used as out-of-bag samples. In order to visualize our spatially explicit predictions of WNV prevalence across the study region, we predicted the prevalence at 20,000 random points in our study area using the relationships between WNV prevalence and predictor variables as determined by random forest. To interpolate a continuous surface among these points, we used both a deterministic (Inverse Distance Weighted) and a geostatistical interpolation method (Ordinary Kriging [53] with linear and spherical models to describe the semivariance [54]), which all resulted in qualitatively comparable surface estimates. Predictions from random forest models were confirmed using Generalized Dissimilarity Modeling (see supporting information Methods Text S1, Table S2), which produced comparable patterns of prevalence across the study area.

To model the spatial distribution of WNV infections in humans across Orange County and to identify the associated environmental or economic variables, we used Maxent (Version 3.1.0), a machine learning algorithm, which has previously been used for modeling of species distributions [55]. Maxent is a general-purpose algorithm that generates predictions or inferences from an incomplete set of information. The Maxent approach is based on a probabilistic framework. The main assumption is that the incomplete empirical probability distribution (determined by occurrence data) can be approximated with a probability distribution of maximum entropy (the Maxent distribution) subject to certain environmental constraints, and that this distribution approximates the potential geographic distribution of the group of interest [56]. The input data consist of a set of environmental layers for the study region and the observed case-presence localities within that region. Maxent then uses these data to build a distribution of the niche space observed at the presence localities, and estimate the environmental properties that are suitable for the taxonomic unit studied. Predictive maps generated by Maxent express suitability of each grid cell as a function of the environmental variables at that grid cell. A high value of the function (in units of logistic probability) at a particular grid cell indicates that the grid cell is predicted to have suitable conditions for the studied unit [56]. Maxent runs with presence-only point occurrences and performs well with few point localities [55]. As a consequence, in a recent large model intercomparison project with 15 other algorithms, Maxent's performance was generally rated among the highest [57]. We modeled WNV infections in humans using the provided presence records throughout the study area with the following predictor variables at 15 m resolution: per capita income, population density, density of neglected pools in the study year, NDVI, elevation, temperature, and the ASTER visible bands. We used the default settings of Maxent: 10,000 background points; linear and quadratic hinge features; regularization multiplier  = 1.0; maximum iterations  = 500; convergence threshold  = 0.00005. To assess the importance of each predictor variable, we ran the jackknifing procedure implemented in Maxent. The area under the receiver operator curve (AUC), implemented in Maxent, was used to assess overall model performance, where an AUC score of 0.5 indicates random prediction, and a score of 1 a perfect prediction. In order to assess the robustness of the model to sampling variation, we used data from 2008 and ran twenty additional models with 75% training and 25% test sites that were randomly selected from the original dataset. We compared the AUC scores of these test runs to that of the full model to determine if any major deviations (i.e. low model performance, with AUC values <0.8) were present that would suggest sensitivity to sampling variation. Except for 2004, for which the random forest model performed poorly compared to the other years, ecological niche models of WNV infections in humans showed a spatial pattern that was highly concordant with prevalence levels of WNV in mosquitoes (Figures 3, S5).

## Supporting Information

Text S1 (DOC)

Figure S1 Average monthly ground temperature in Orange County, California, for years 2004-2006. Shaded areas represent months for which West Nile virus data was collected. Temperature differences between nine ground stations across the study area were fairly consistent across months and across years; thus, heterogeneity in surface kinetic temperatures recorded in May (the beginning of each sampling period) were used as a surrogate for the spatial heterogeneity seen across the study area for the entire sampling period. (TIF)

Figure S2 Average monthly precipitation in Orange County, California, for years 2004-2006. Shaded areas represent months for which West Nile virus data were collected. Little precipitation fell during the months for which West Nile virus data were collected, warranting an exploration of artificial and standing water sources in our analyses. (TIF)

Figure S3 Spatial autocorrelation results for prevalence levels of WNV in mosquitoes, as measured by the Maximum Likelihood Estimate. Blue lines indicate the autocorrelation coefficient *r*, red lines indicate 95% confidence levels of 999 randomizations of sampling localities and bars indicate 95% confidence levels of 1000 bootstrap replicates. Negative correlations suggest that similar MLE values are more dispersed than expected at random. (TIF)

Figure S4 Tree regression results for WNV prevalence in vectors. At each node, the splitting variable for that node is indicated. The branch left of the node represents lower values for the splitting variable, whereas the branch right of the node represents higher values. Figures at the terminal ends indicate prevalence levels (as measured by maximum likelihood estimates). (TIF)

Figure S5 Model predictions for WNV prevalence in vectors and humans for 2004, 2005, and 2008. Predictions in vectors are based on random forest models, whereas Maxent was used to predict WNV in humans. Colors indicate the relative prevalence in vectors and probability of human cases within each year (see color bars). Colors for the predicted WNV prevalence in vectors are scaled for each year to span the entire range of predicted prevalence levels in the corresponding year. Scale bar is an approximation, as scale varies according to perspective. (TIF)

Figure S6 Jackknifing results to test for variable importance in Maxent models for the distribution of WNV infections in humans in 2004, 2005, and 2008. Light blue bars indicate model performance when the variable is omitted. Dark blue bars indicate model performance when the variable is used by its own. Blue, green, red  =  visual ASTER bands; elevation  =  ASTER digital elevation model (DEM) at 25 m resolution; NDVI  =  Normalized Difference Vegetation Index; neglected pools  =  neglected swimming pools in the study year; income  =  per capita income; population density  =  human population density; temperature  =  surface kinetic temperature measured by ASTER. (TIF)

Table S1 (DOC)

Table S2 (DOC)
