# Supplementary material for: Economic Conditions Predict Prevalence of West Nile Virus
Source: PLoS One. 2010 Nov 12;5(11):e15437. doi: 10.1371/journal.pone.0015437 (PMC2980475; doi:10.1371/journal.pone.0015437)
Supplement: Text S1 — (DOC) [file pone.0015437.s001.doc]

**Supporting Information**

**Economic Conditions Predict Prevalence of West Nile Virus**

Ryan J. Harrigan, Henri A. Thomassen, Wolfgang Buermann,

Robert F. Cummings, Matthew E. Kahn, Thomas B. Smith

**Materials and Methods**

**Spatial autocorrelation.** Spatial autocorrelation statistics measure the effect of proximity of sampling sites on the variable of interest. This effect could either be positive, where nearby sites are more similar to each other than expected by chance, or negative, where nearby sites are more different than expected by chance. In order to assess the potential influence of spatial proximity on prevalence levels, we calculated Moran’s I (S1) in ArcMap 9.2 and the autocorrelation coefficient *r* (S2) in Genalex (S3). The autocorrelation coefficient *r* was calculated and tested for significance using 999 permutations and 1000 bootstrap replicates to estimate 95% confidence intervals for the effects of randomizing the spatial structure of the entire dataset and taking a subsample of the full dataset respectively (S3).

**Spatial modeling.** In addition to random forest models, we verified spatial predictions using generalized dissimilarity modeling (GDM) (S4), which models spatial variation of response variables across a heterogeneous landscape. GDM is a matrix regression technique, employing I-spline basis functions, which allows for fitting non-linear relationships of environmental variables to biological variation. A major advantage of GDM over other modeling methodologies is that it can explicitly and simultaneously take into account the influence of both geographic distance (i.e. spatial autocorrelation) and environmental variables on explaining biological variation. It is a two-step method: first, dissimilarities of a set of predictor variables are fitted to the dissimilarities in prevalence levels (the response variables). Predictor contributions to explaining the observed response variation are tested by Monte-Carlo permutation, and only those that are significant are retained in the final model. These procedures result in a function that describes the relationship between environment and response variables. Subsequently, using this resulting function, a spatial prediction is made of the response variable patterns. GDM provides information on the relative importance of predictor variables by means of response curves. Thus, the influence of geographic distance relative to other variables in explaining variation in prevalence levels can be assessed even when localities of similar prevalence levels are spatially autocorrelated. To further assess the extent to which geographic distance is potentially correlated with other predictor variables, we ran independent tests with the following sets of predictor variables: 1) environmental and socio-economic variables and geographic distance; 2) only geographic distance; 3) only environmental and socio-economic variables. When both 2) and 3) explain a high percentage of the total variation, caution should be taken in interpreting the results from 1), because in that case geographic distance is highly correlated with differences in environmental and socio-economic factors. Because no formalized significance testing has yet been developed for GDM, in order to assess the significance of the level of variation that was explained by our models, we ran additional models in which the environmental and economic layers were substituted by layers with random values for each grid cell. The resulting percentage of variation explained was subsequently compared to that of the full model. We considered the performance of the full model not significant if it explained an equal or less amount of the total variation than a model with random environmental variables.

**Results**

**Spatial Autocorrelation.** For all years, WNV prevalence in vectors was either not significantly clustered, or significantly clustered, but with low to medium values for Moran’s I (Table S1) and *r* (Fig. S3). Further assessment of the distance within which samples showed clustering indicated that only those localities that were close to each other (i.e. within 10% of the maximum distance between any two sites) were more similar than expected by chance (Fig. S3). More than 90% of the pairwise comparisons were at distances where spatial autocorrelation could not be detected. Because of this very limited effect of spatial autocorrelation, we did not correct for spatial proximity in subsequent analyses. In addition, removing the effect of spatial proximity would also partially remove the effect of environmental and socio-economic variables, because these variables are by their nature spatially structured. Our approach of not removing spatial autocorrelation from further analyses was supported by the fact that generalized dissimilarity models, which explicitly take into account and test for the effect of distance, did not select geographic distance as a variable significant in explaining the observed variation in prevalence levels (see below).

**Spatial Modeling.** Generalized dissimilarity models for WNV prevalence in vectors in 2004, 2005, and 2008 explained a maximum of 47.1% of total observed variation, and all models performed considerably better than those with random environmental variables (Table S2). The density of neglected swimming pools and per capita income were consistently significant predictor variables across all study years, whereas geographic distance was not significant in any of the models. The predictive maps of WNV prevalence in vectors were highly concordant with those from the random forest, and we therefore present only the latter. Regression trees for WNV prevalence in vectors showed that per capita income consistently explained the most variation of any predictor variable across all three study years (Fig. S4), as indicated by the branch lengths. Other variables were also included in the regression trees, but these only explained a relatively small amount of the variation, and were not consistent across years.

**Supporting Information References**

S1. Moran PAP (1950) Notes on continuous stochastic phenomena. *Biometrika* 37:17–33.

S2. Peakall R, Ruibal M, Lindenmayer DB (2003) Spatial autocorrelation analysis offers new insights into gene flow in the Australian bush rat, *Rattus fuscipes*. *Evolution* 57:1182-1195.

S3. Peakall R, Smouse PE (2006) GENALEX 6: genetic analysis in Excel. Population genetic software for teaching and research. *Mol. Ecol. Notes* 6:288-295.

S4. Ferrier S, Manion G, Elith J, Richardson K (2007) Using generalized dissimilarity modelling to analyse and predict patterns of beta diversity in regional biodiversity assessment. *Divers Distrib* 13:252-264.
