# Supplementary figures and images for: Economic Conditions Predict Prevalence of West Nile Virus
Source: PLoS One. 2010 Nov 12;5(11):e15437. doi: 10.1371/journal.pone.0015437 (PMC2980475; doi:10.1371/journal.pone.0015437)

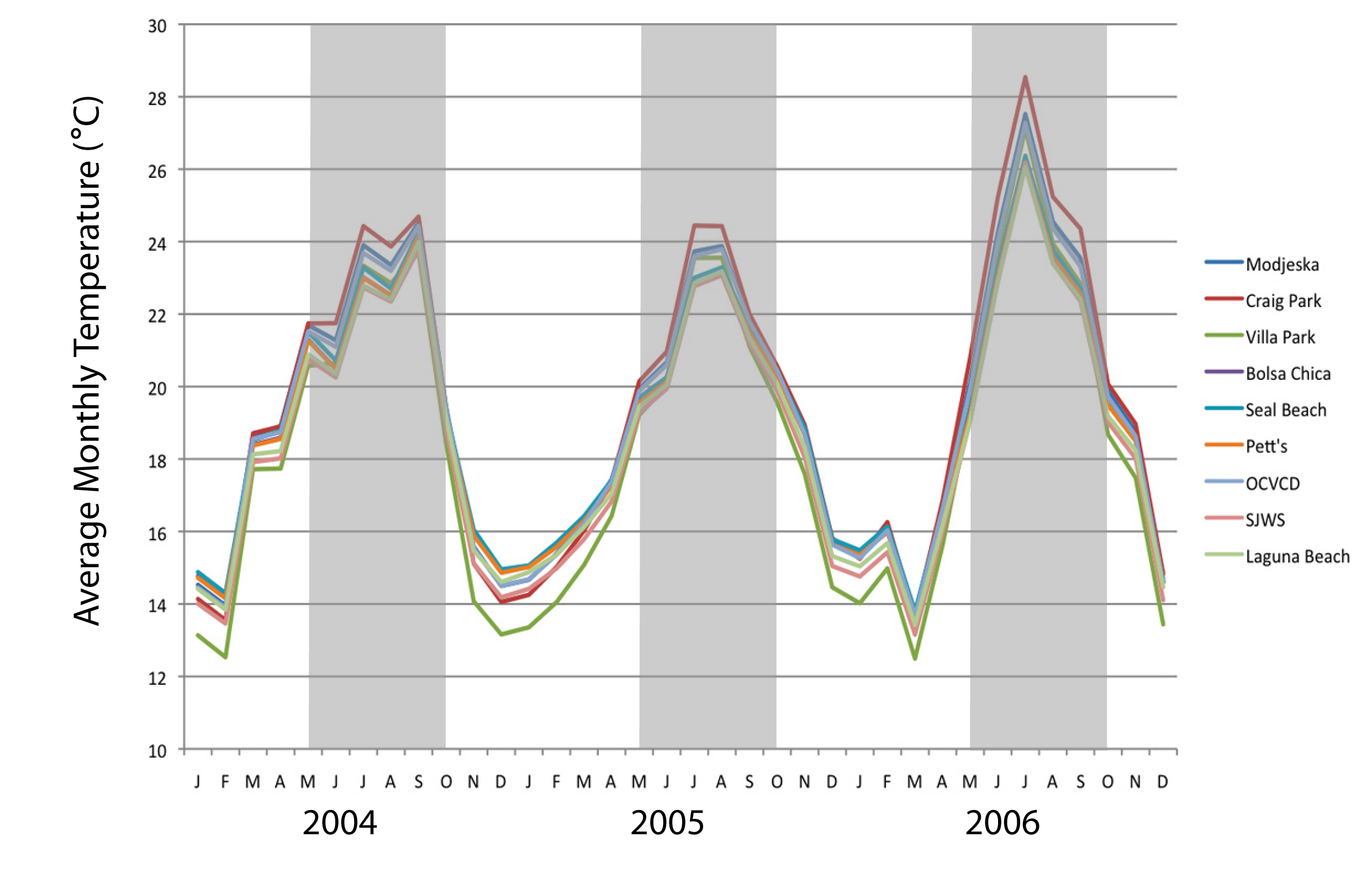

Supplement: Figure S1 — Average monthly ground temperature in Orange County, California, for years 2004-2006. Shaded areas represent months for which West Nile virus data was collected. Temperature differences between nine ground stations across the study area were fairly consistent across months and across years; thus, heterogeneity in surface kinetic temperatures recorded in May (the beginning of each sampling period) were used as a surrogate for the spatial heterogeneity seen across the study area for the entire sampling period. (TIF) [file pone.0015437.s002.tif]

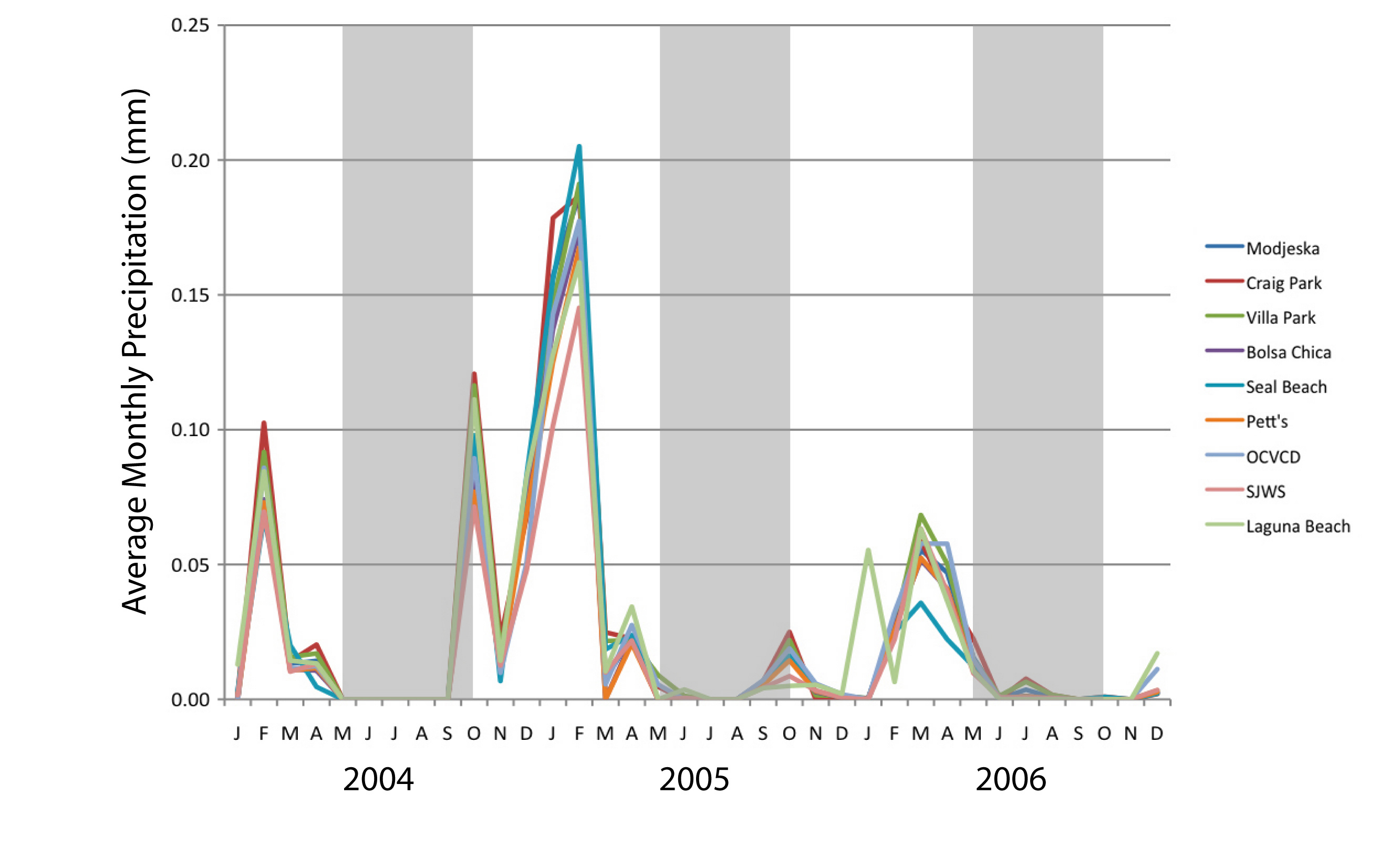

Supplement: Figure S2 — Average monthly precipitation in Orange County, California, for years 2004-2006. Shaded areas represent months for which West Nile virus data were collected. Little precipitation fell during the months for which West Nile virus data were collected, warranting an exploration of artificial and standing water sources in our analyses. (TIF) [file pone.0015437.s003.tif]

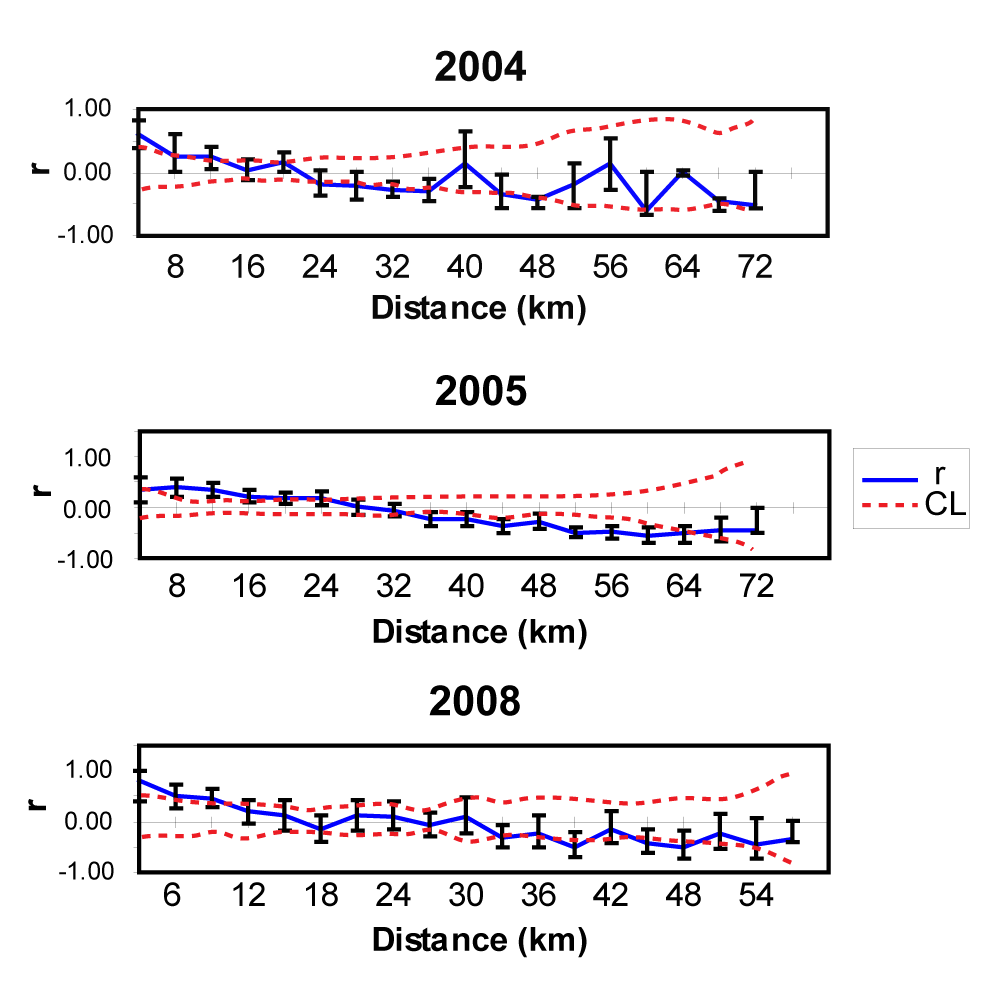

Supplement: Figure S3 — Spatial autocorrelation results for prevalence levels of WNV in mosquitoes, as measured by the Maximum Likelihood Estimate. Blue lines indicate the autocorrelation coefficient r, red lines indicate 95% confidence levels of 999 randomizations of sampling localities and bars indicate 95% confidence levels of 1000 bootstrap replicates. Negative correlations suggest that similar MLE values are more dispersed than expected at random. (TIF) [file pone.0015437.s004.tif]

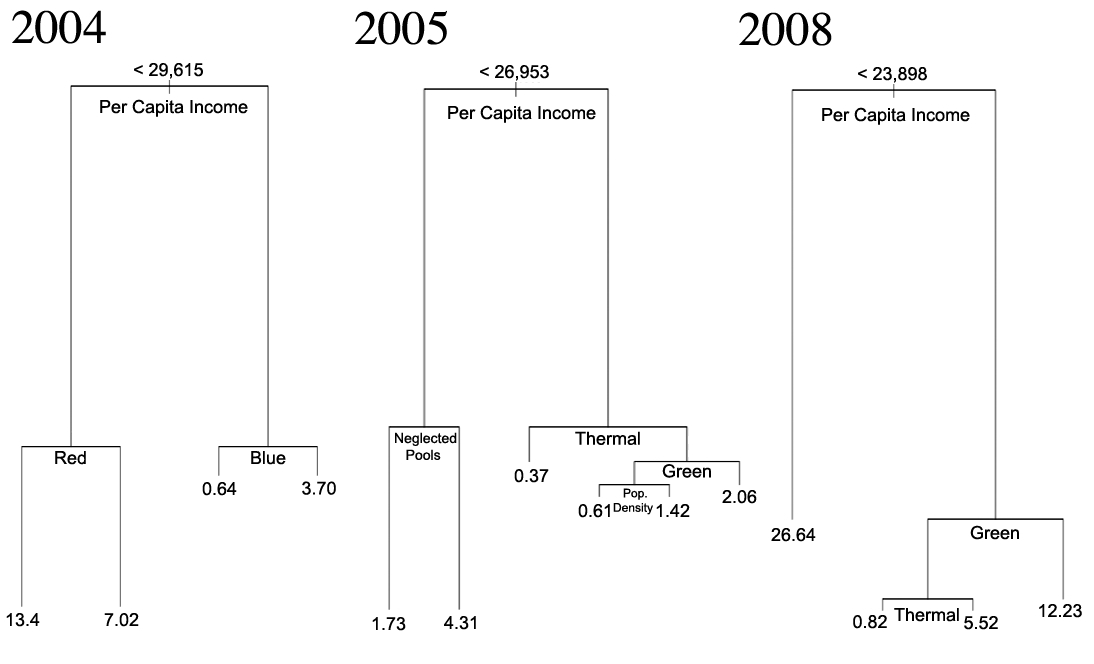

Supplement: Figure S4 — Tree regression results for WNV prevalence in vectors. At each node, the splitting variable for that node is indicated. The branch left of the node represents lower values for the splitting variable, whereas the branch right of the node represents higher values. Figures at the terminal ends indicate prevalence levels (as measured by maximum likelihood estimates). (TIF) [file pone.0015437.s005.tif]

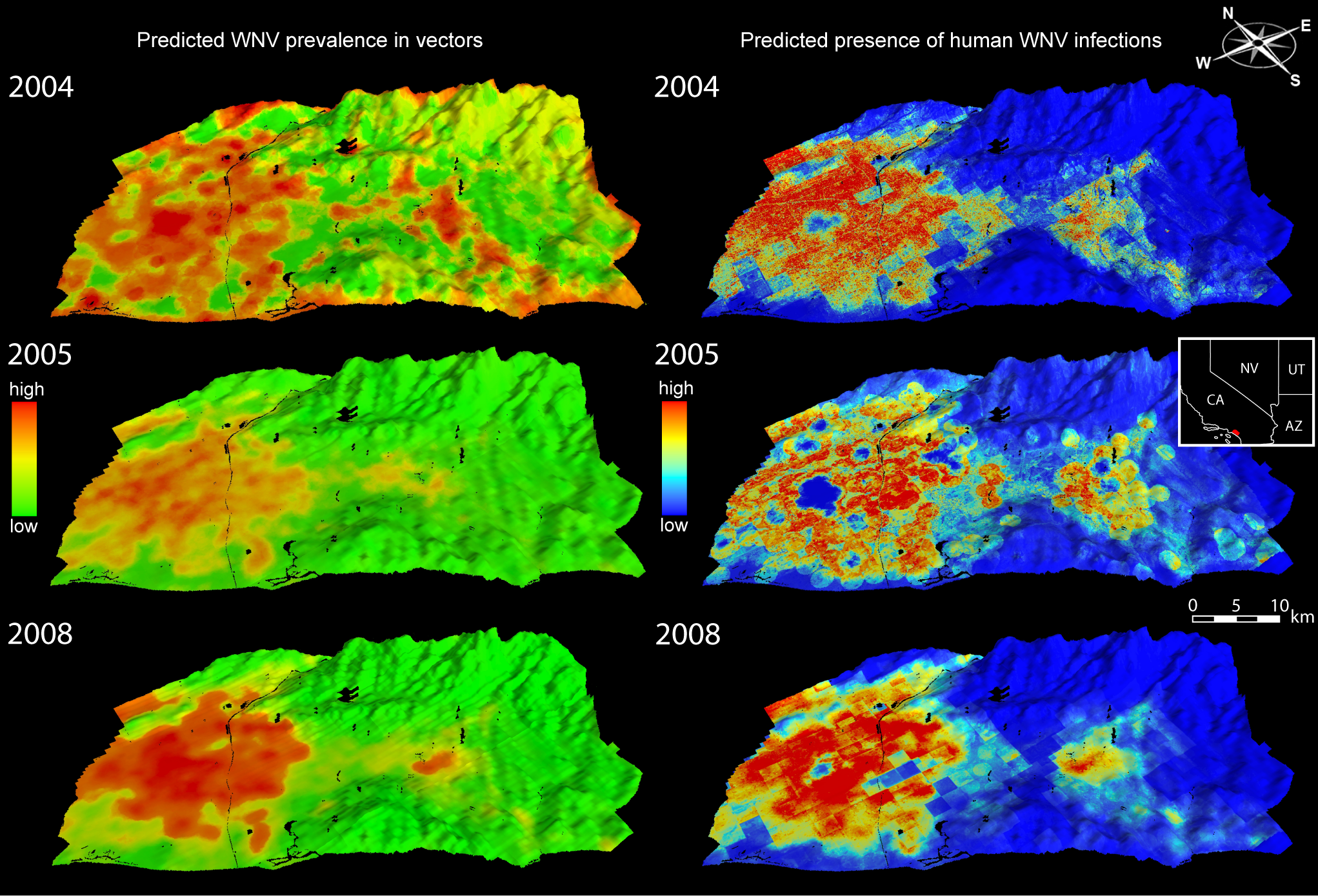

Supplement: Figure S5 — Model predictions for WNV prevalence in vectors and humans for 2004, 2005, and 2008. Predictions in vectors are based on random forest models, whereas Maxent was used to predict WNV in humans. Colors indicate the relative prevalence in vectors and probability of human cases within each year (see color bars). Colors for the predicted WNV prevalence in vectors are scaled for each year to span the entire range of predicted prevalence levels in the corresponding year. Scale bar is an approximation, as scale varies according to perspective. (TIF) [file pone.0015437.s006.tif]

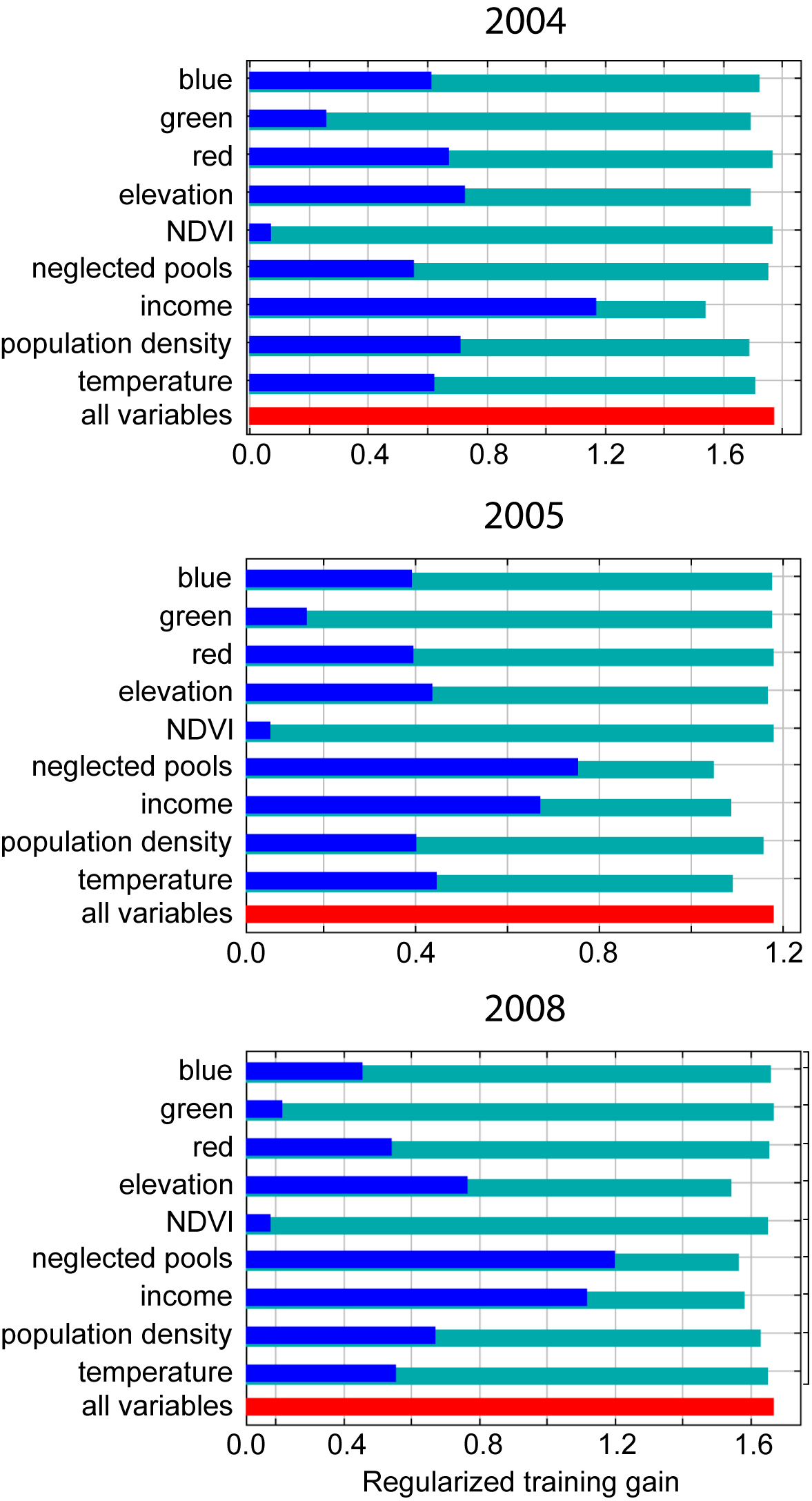

Supplement: Figure S6 — Jackknifing results to test for variable importance in Maxent models for the distribution of WNV infections in humans in 2004, 2005, and 2008. Light blue bars indicate model performance when the variable is omitted. Dark blue bars indicate model performance when the variable is used by its own. Blue, green, red = visual ASTER bands; elevation = ASTER digital elevation model (DEM) at 25 m resolution; NDVI = Normalized Difference Vegetation Index; neglected pools = neglected swimming pools in the study year; income = per capita income; population density = human population density; temperature = surface kinetic temperature measured by ASTER. (TIF) [file pone.0015437.s007.tif]
