# Supplementary material for: Economic Conditions Predict Prevalence of West Nile Virus
Source: PLoS One. 2010 Nov 12;5(11):e15437. doi: 10.1371/journal.pone.0015437 (PMC2980475; doi:10.1371/journal.pone.0015437)
Supplement: Table S1 — (DOC) [file pone.0015437.s008.doc]

|  | 2004 | 2005 | 2008 |
| --- | --- | --- | --- |
| I | 0.125 | 0.243 | 0.364 |
| Z | 1.163 | 7.196 | 3.852 |
| p | >0.1 | **<0.05** | **<0.05** |
