# Supplementary material for: Economic Conditions Predict Prevalence of West Nile Virus
Source: PLoS One. 2010 Nov 12;5(11):e15437. doi: 10.1371/journal.pone.0015437 (PMC2980475; doi:10.1371/journal.pone.0015437)
Supplement: Table S2 — (DOC) [file pone.0015437.s009.doc]

| Year | Percentage of variance explained (full model / only distance / only environment and economy / random) | Significant variables in full model |
| --- | --- | --- |
| 2004 | 25.1 / 0 / 25.1 / 1.7 | dens. negl. sw.pools; blue; pc income; temp |
| 2005 | 18.5 / 0 / 18.5 / 1.7 | dens. negl. sw.pools; pc income; red; temp; green |
| 2008 | 47.1 / 0 / 47.1 / 0.5 | dens. negl. sw.pools; pc income; temp; green; red |

dens. negl. sw.pools = density of neglected swimming pools in a 1 km range; pc income = per capita household income; temp = temperature measured by ASTER; blue = blue visual band from ASTER; green = green visual band from ASTER; red = red visual band from ASTER
